# Shining a Spotlight on DNA: Single-Molecule Methods to Visualise DNA

**DOI:** 10.3390/molecules24030491

**Published:** 2019-01-30

**Authors:** Gurleen Kaur, Jacob S. Lewis, Antoine M. van Oijen

**Affiliations:** 1 School of Chemistry and Molecular Bioscience and Molecular Horizons, University of Wollongong, Wollongong 2522, Australia; gk980@uowmail.edu.au (G.K.); jacobl@uow.edu.au (J.S.L.); 2 Illawarra Health and Medical Research Institute, Wollongong, New South Wales 2522, Australia

**Keywords:** DNA, single molecules, fluorescence microscopy, DNA complexes, protein complexes

## Abstract

The ability to watch single molecules of DNA has revolutionised how we study biological transactions concerning nucleic acids. Many strategies have been developed to manipulate DNA molecules to investigate mechanical properties, dynamics and protein–DNA interactions. Imaging methods using small molecules and protein-based probes to visualise DNA have propelled our understanding of complex biochemical reactions involving DNA. This review focuses on summarising some of the methodological developments made to visualise individual DNA molecules and discusses how these probes have been used in single-molecule biophysical assays.

## 1. Introduction

The development of physical tools to study biological processes on DNA with high resolution has yielded unprecedented insight into the molecular mechanisms that define life. An array of methods have been developed to allow for the visualisation and manipulation of individual DNA molecules. Many of these advancements have been driven by our quest to understand how DNA is read, copied, stored and protected inside cells. Progress through technical innovations and multi-disciplinary research efforts combining chemistry, molecular biology and microscopy have provided detailed snapshots of the inherent molecular structure, dynamics and roles DNA adopts during biochemical reactions. In particular, studies of DNA replication have benefited greatly from single-molecule fluorescence microscopy, where information obtained from traditional methodologies is combined with single-molecule imaging. This marriage of information has led to many significant breakthroughs and subsequently has challenged our understanding of how these multi-protein complexes operate on DNA.

To introduce this review, first, a few words on why single-molecule DNA studies are important, especially given the considerable effort required to carry them out. So why study DNA molecules with single-molecule sensitivity when it is often easier to study averaged populations in solution-phase experiments? It is clear that a wealth of knowledge may be extracted from ensemble-based methods examining the averaged properties of DNA. However, detailed understanding of short-lived intermediate states in complex processes or rare events simply cannot be gained by looking at an ensemble of DNA molecules. Further, it is not possible to directly examine the dynamics of DNA transitioning from an inactive state/complex to an active state/complex when only a small fraction of molecules exist in a particular state at a given moment. Only single-molecule-based approaches are able to probe the heterogeneity of molecular behaviours across a population without the need to trap intermediate states, synchronise reactions or infer transitions from one discrete conformation to another.

Over the last two decades, a variety of single-molecule techniques have been developed to examine DNA in isolation and within the context of biological processes. These techniques include: atomic force microscopy [1], magnetic tweezers [2], optical monitoring of fluorescent probes [3,4], single-molecule fluorescence resonance energy transfer (smFRET) [5] and tethered-bead flow stretching [6]. Each technique has limitations, whether it is throughput, time resolution, or the modifications of DNA required to visualise particular processes or conversions between states. More often than not, these limitations are inherent to the physical or temporal selectivity needed in order to resolve single DNA molecules and adequately study them. Generally, this means localising DNA in a discrete volume, so it can be physically separated and distinguished from other molecules. Localisation is frequently carried out by attachment of DNA to a solid surface (i.e., beads, glass or proteins) and confinement to a microfluidic channel controlled by flow, electrochemical gradients or lasers. Moreover, depending on the time resolution of the experiment or type of data being collected, (i.e., real-time, staged or end-point), it is important to precisely track individual target molecules over time. In doing so, detailed information can be extracted from single-molecule time trajectories at a level of detail that is otherwise hidden in conventional ensemble studies. Access to these particular details has been made possible through the development of sensitive electron-multiplying charge-coupled device cameras, software/algorithm development, improvement of conjugation chemistry, preparation of new fluorescent chemical compounds and reconstitution of complex multi-protein complexes.

**Figure 1:**
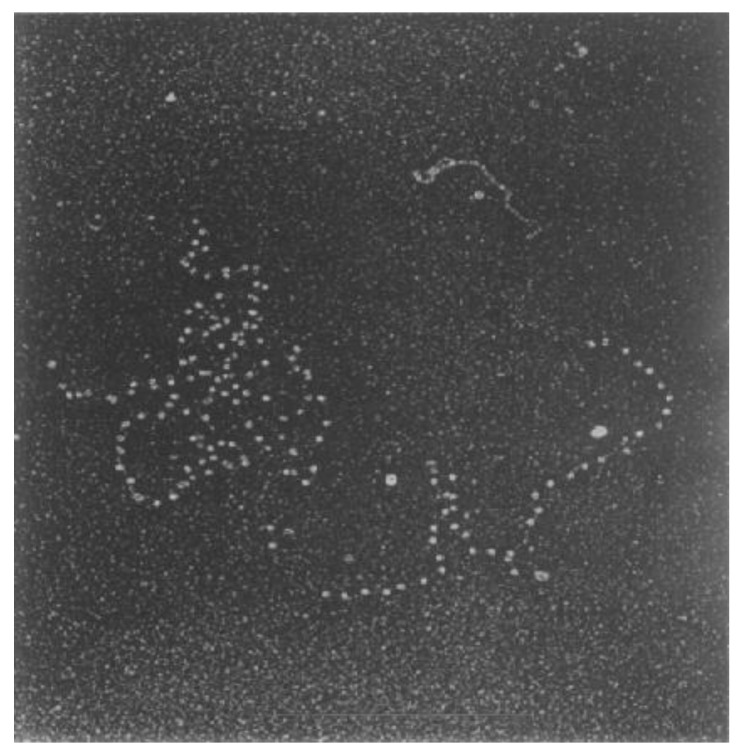
Visualising DNA molecules using electron microscopy. An electron micrograph generated by shadow-casting electron microscopy of multiple DNA polymerase proteins bound to individual DNA molecules (reproduced with permission from [11]).

After the publication of the structure of the double helix [7,8], attention was focused on understanding the role of nucleic acids in biological processes, specifically those that use DNA as a template. It was not surprising that these efforts resulted in methodological advancements to achieve the direct visualisation of single DNA molecules. Electron microscopy (EM) was at the forefront of these methodological developments. Building on work from William and Wyckoff [9], Hall and later Griffith established shadow-casting EM as an ideal technique to visualise individual DNA molecules in isolation and bound to specific protein complexes [10,11] (Figure 1). Intensive research using EM over the next two decades resulted in numerous high-resolution DNA structures. Through interpretation of these high-resolution static snapshots, researchers realised that DNA molecules vary in structure across whole populations and that DNA exists in different types of shapes and topological forms, depending on its origin and what biological processes were acting on it. Although continued progress and tool development in EM has rapidly expanded in the intervening thirty years, the challenges remain the same. How can we select individual DNA molecules and study their behaviours to better understand the structures and dynamics across entire populations? Furthermore, how do we reconcile these behaviours in the context of complex biochemical reactions, such as DNA replication?

**Figure 2:**
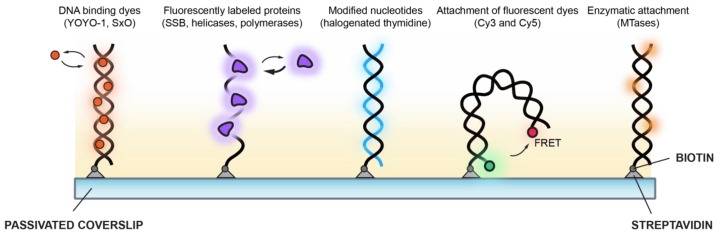
Overview of different fluorescent probes developed to detect single DNA molecules using single-molecule fluorescence microscopy. (From left to right) DNA binding dyes such as YOYO-1 and SYTOX Orange (SxO) remain largely non-fluorescent in solution and become highly fluorescent upon interaction with the bases in DNA, enabling direct visualisation during complex biochemical reactions. Fluorescently labelled proteins such as single-stranded binding proteins (SSB) provide a method to visualise long pieces of single-stranded DNA. Modified nucleotides such as 5-bromo-2-deoxyuridine can be incorporated directly into the newly synthesised DNA and labelled by treatment with fluorescently labelled antibodies. Fluorescent dye molecules such as Cy3 and Cy5 can be installed at specific positions in the DNA used in single-molecule fluorescence resonance energy transfer (smFRET) studies to monitor conformational dynamics. DNA methyltransferases (MTases) are able to recognise specific sequences within DNA and covalently link fluorescently labelled cofactors to generate high-resolution optical maps of large DNA fragments.

Visualisation of DNA and the transactions that occur on it by single-molecule approaches has expanded so rapidly that it is not possible to comprehensively review all of it. Therefore, with a focus on single-molecule fluorescence methods to visualise DNA, this review will use a small number of representative areas of study to explore the development and evolution of the impressive toolkit that has been developed to understand the dynamics of DNA. Moreover, we will contextualise these fluorescence-based tools in studying the molecular details fundamental to DNA replication. These toolkits are broadly divided into those which detect single DNA molecules using the binding or attachment of small molecules and those which utilise enzymatic activity or binding (Figure 2).

## 2. Visualisation of DNA Dynamics and Topological Intermediates

Single-molecule fluorescence microscopy has allowed unprecedented examination of the dynamics of DNA in solution. By coupling fluorescence microscopy with the ability to localise individual DNA molecules, many unanswered questions in DNA polymer dynamics and topology have been revisited. As DNA itself has no fluorescent properties that can be exploited for single-molecule detection, it must be “stained” to be detected. Typically, fluorescent ligands that intercalate between base pairs or bind into the helical grooves are used. The first real-time visualisation of single DNA molecules, carried out by Morikawa and Yanagida [12] by staining the double-stranded (ds) DNA isolated from T4 bacteriophage with DAPI, allowed for different structural transitions to be observed. However, the poor spectroscopic and binding properties of standard dye molecules such as DAPI made it difficult to obtain high-quality images of single DNA molecules. The development and commercial production of new dsDNA-binding dyes that remained largely non-fluorescent in solution and displayed large increases in their fluorescent quantum yield upon binding DNA have revolutionised single-molecule imaging of DNA. Of particular note are two dye molecules: YOYO-1 and SYTOX Orange (SxO). Both YOYO-1 and SxO have transformed our capacity to carry out detailed single-molecule studies of DNA, both in isolation and within complex biochemical reactions. Therefore, in the following paragraphs we will describe the properties of YOYO-1 and SxO that make them invaluable to studying DNA at the single-molecule level. Moreover, we will discuss milestone studies that utilised YOYO-1 and SxO during investigations of DNA dynamics and topological intermediates.

### 2.1. YOYO-1

YOYO-1, a dimeric bis-intercalator, was rapidly adopted by the single-molecule biophysics community due to its attractive kinetic and fluorescence properties. YOYO-1 is a cyanine dye emitting fluorescence in the green portion of the spectrum. YOYO-1 is largely non-fluorescent in solution and interacts with dsDNA with high affinity (*K*_D_ = 5–50 nM, depending on ionic strength) [13,14,15]. Binding to dsDNA results in a 1000-fold increase in fluorescence intensity. The subsequent increase in the signal-to-background ratio allows for easier detection of DNA molecules. Since the development of YOYO-1 by Rye et al. [16], the dye has been successfully used to study dynamics of individual molecules of phage genomic DNA in solution [17] and the mechanisms of motion of large DNA molecules during constant-field and pulsed-field gel electrophoresis [18], as well as visualising the contour lengths of elongated DNA [19]. The use of YOYO-1, however, is not always fully compatible with all experimental conditions. While binding tightly to dsDNA, YOYO-1 requires long incubation times and high temperature to obtain homogeneous DNA staining [20]. In experimental designs geared towards single-molecule fluorescence real-time imaging, the dye binds rather slowly to individual DNA molecules (~5 min for every 40 nM of YOYO-1) [14]. Like all intercalating fluorescent dyes, photocleavage of the DNA as a direct result of irradiation with laser light during single-molecule imaging represents a challenge [21,22,23]. This type of DNA damage occurs once the fluorophores are excited, as they may undergo intersystem crossing, generating free radicals such as reactive oxygen species (ROS). These free radicals can then attack the DNA phosphate backbone to produce various forms of oxidative photo damage, resulting in single-strand breaks. It is the accumulation of single-strand breaks in the DNA that can lead to double-strand breaks and loss of the DNA from the surface in single-molecule experiments. Consequently, imaging chemistries are continuously being developed to mitigate this effect, by designing ROS scavenging systems and new fluorescent probes [24]. A successful approach is enzymatic systems that reduce the concentration of oxygen in solution, such as glucose oxidase/catalase [25], protocatechuic acid/protocatechuate-3,4-dioxygenase [26] and pyranose oxidase/catalase [27].

### 2.2. SYTOX Dyes

SYTOX dyes were originally designed and marketed for staining dead cells and come in a range of colours [28]. More recently, SYTOX dyes have been used for single-cell and single-molecule fluorescence experiments [23,29,30,31]. In particular, SxO, a cyanine dye, has rapidly become the gold standard for visualising DNA at the single-molecule level. While the exact structure of SxO is proprietary, it is reported to be monomeric and to intercalate when binding to dsDNA [32]. SxO has several key advantages over other fluorescence DNA dyes such as YOYO-1. SxO exhibits a >1000-fold increase in fluorescence upon binding dsDNA, while showing little base selectivity. SxO also has relatively high binding and dissociation rates [33,34], allowing equilibrium to be achieved immediately after introduction of the dye to the DNA. The high binding and dissociation rates also minimise the number of photobleached dye molecules bound to DNA, as photobleached dye molecules will quickly disassociate and get replaced. These properties make SxO (and SxG [24]) superior to YOYO-1 when measuring fluctuations or topological changes in DNA.

### 2.3. Polymer Physics with Single DNA Molecules

DNA is frequently used as a model system to study physical principles of polymer behaviour as it is large enough to visualise its shape at the single-molecule level, yet small enough such that thermal fluctuations dominate its motion. Intensive research over many decades resulted in numerous mathematical models to describe the concentration-dependent arrangement of polymers in solution and the snake-like motion of polymers, aptly termed reptation [35,36]. It was not until a series of landmark single-molecule studies were conducted by the Chu lab in the 1990s that a number of important models and hypotheses concerning the elastic and dynamic properties of DNA were experimentally tested and verified [37]. These experiments relied on YOYO-1 to stain individual DNA molecules bound to microbeads, which were then simultaneously manipulated by optical tweezers. By precisely tracking the molecular fluctuations of individual DNA molecules, Chu and colleagues were able to directly observe the tube-like motion of DNA in entangled solutions of DNA [38]. These observations were critical in establishing the idea of “molecular individualism”, where identical molecules in the same initial state will choose several distinct pathways to a new equilibrium state [39,40]. Finally, using a cross-slot microfluidic chamber, Schroeder and co-workers [29] were able to track the equilibrium extension of individual DNA molecules in extensional flow and characterise their viscoelastic properties. This work enabled the first direct observation of polymer conformation hysteresis.

### 2.4. Knotted DNA

DNA of short length (less than the persistence length of ~50 nm) is a very stiff polymer. However, in cells where it is present in much longer forms (up to many centimetres), it displays a much larger degree of flexibility and conformational freedom, making it highly susceptible to self-entanglement and knotting [41,42,43]. Knots in DNA can occur as byproducts of fundamental biological processes, such as transcription, DNA replication, recombination, topoisomerisation and compaction [44,45,46,47].

**Figure 3:**
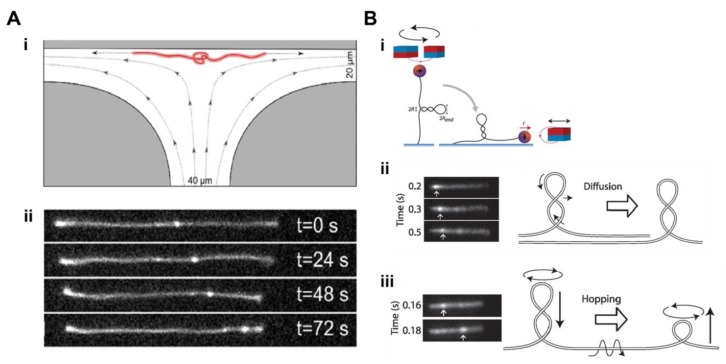
Fluorescence visualisation of topological intermediates of DNA. (**A**) Motion of knots in DNA: (**i**) A microfluidic T-junction flow cell with a diverging electric field stretches knotted linear DNA molecules at its stagnation point. (**ii**) Representative images of a single DNA molecule at four time points as a DNA knot (bright fluorescent spot) translates towards one end of the DNA molecule (reproduced with permission from [56]). (**B**) Dynamics of DNA supercoils: (**i**) Visualisation of plectonemes by fluorescence microscopy combined with magnetic tweezers. Individual DNA molecules are supercoiled by rotating a pair of magnets and subsequently pulled sideways by another magnet. (**ii**) Fluorescence images of plectoneme diffusion along an individual supercoiled DNA molecule stained with SxO. (**iii**) Fluorescence images of a plectoneme hopping along an individual supercoiled DNA molecule stained with SxO (reproduced with permission from [66]).

Production of DNA knots has been achieved in vitro using high electric fields [48], topoisomerase enzymes [46,49] DNA recombinases [44,50], cyclisation of linear DNA molecules, and most notably, through manipulation by optical tweezers [51,52]. Traditionally, EM has been used to image knots with high resolution. Thus, analysis of EM micrographs has allowed visualisation of numerous, well-defined types of DNA knots generated under various conditions within a product population [53,54]. While EM enabled detailed studies into the types of DNA knots present under various conditions, no information concerning the mobility of DNA knots was available. Using YOYO-1 stained bead–DNA–bead dumbbells manipulated by optical tweezers, Bao and colleagues developed a system capable of tying several knots into individual DNA molecules and observing their dynamic behaviour, one knot at a time [52]. Mechanically knotting DNA with beads at either end meant the DNA was kept under a fixed tension, making localised knot properties independent of the length of DNA. By directly imaging confined DNA molecules containing knots, they demonstrated that knots are able to diffuse via a reptation mechanism. Moreover, the measured knot diffusion constants were correlated with knot complexity. More recently, however, studies aimed to understand the motion of knots along DNA has intensified, growing with the development of DNA sequencing applications that enable reads several tens of thousands of bases long. Given the technical challenges associated with quantifying knot mobility at the nanoscale, many of these studies have been computer simulations [55]. Recently, however, direct quantification of knot motions on unmodified single DNA molecules has been reported. Using T-shaped microfluidic channels and a divergent electric field, Klotz and colleagues were able to create knotted DNA with different topologies and track their movement over time [56] (Figure 3A). This novel experimental approach confirmed previous theoretical predications that DNA knots are able to diffuse along uniformly stretched chains, be driven towards the ends of the molecule and untie [57]. Moreover, by increasing the electric field, thus increasing tension, knot diffusion slowed. At higher tensions (Weissenberg number = 1.9), the knots jammed, possibly through intramolecular friction [56,58]. Overall, these results are consistent with previous studies showing knots moving along nanochannel-confined DNA [59] and knots sliding along DNA as they translocated through nanopores [60].

### 2.5. DNA Supercoiling

Today, we understand that DNA is topologically polymorphic, that is, it can exist in many different structural forms. One of these forms, the supercoil, was first illustrated by Vinograd and co-workers [61] in EM micrographs of circular DNA from polyoma virus. These micrographs revealed the presence of multiple intertwined loops. These loops, also called plectonemes, play an important role in the function of DNA inside the cell; for example, the destabilisation of certain DNA sequences [62] and bringing together of distant DNA loci such as transcriptional enhancers or promotors [63,64]. While high in resolution, the static snapshots generated by EM left many unanswered questions about the dynamics of plectonemes—are they static? How do they nucleate, grow and shrink?

Mechanistic insights into the dynamics of DNA supercoiling and the effects of related DNA-processing enzymes that change the state of supercoiling have remained largely speculative, mainly due to the lack of appropriate experimental tools required to effectively study these dynamic behaviours. Single-molecule magnetic tweezers, however, have proven an ideal experimental approach to study DNA mechanics, as they allow twisting and application of a precise stretching force. However, these single-molecule techniques measure the end-to-end extension of DNA, providing limited structural information. Dynamic manipulation of DNA using magnetic tweezers was first demonstrated by Strick et al. [65], where a DNA molecule was torsionally constrained between a glass slide and a superparamagnetic bead. A pair of magnets pulled the bead vertically toward the magnets and DNA was supercoiled through the rotation of the bead. As the length of DNA decreased with the twisting of the bead, plectoneme nucleation was observed. However, it was not until the coupling of single-molecule magnetic tweezers with fluorescence imaging that the dynamics of plectonemes could be directly observed. Pioneering work by the lab of Dekker resulted in the development of a magnetic tweezers apparatus that pulled on SxO-stained DNA molecules sideways while simultaneously visualising them [66]. In this supercoiled DNA, bright fluorescent spots reflected high local DNA density, consistent with the existence of plectonemes (Figure 3B). Plectonemes moved along DNA by diffusion, or unexpectedly, by a fast ‘hopping’ process that facilitated very rapid long-range plectoneme displacement by nucleating a new plectoneme at a distant position. These findings have important implications for the processes that take place across genomic DNA, such as the regulation of gene transactions, sequence searching during DNA recombination or enhancer-activated gene expression. This novel experimental approach provided a powerful method to visualise and study the dynamics of DNA supercoiling outside the cell.

The complicated instrumentation and sample preparation required to study plectoneme dynamics in real-time with single DNA molecules has presented challenges for the accessibility of these approaches. A high-throughput single-molecule assay for real-time visualisation of supercoiled DNA molecules using a conventional fluorescence microscope, named ISD (intercalation-induced supercoiling of DNA) has largely mitigated these barriers [67]. In this approach, SxO is used to induce supercoiling of linear DNA molecules bound to a surface where the two ends are torsionally constrained. DNA and plectoneme dynamics are visualised by near-TIRF microscopy, and the positions and sizes of individual plectonemes can be characterised. Development of ISD has enabled non-specialist researchers to explore how DNA structure is influenced by DNA sequence and enzymatic activity on supercoiled DNA.

### 2.6. DNA Looping

DNA loops are created when proteins or multi-protein complexes bind to different sites on the same DNA molecule simultaneously. Consequently, the intermediary DNA loops out, resulting in loops potentially up to hundreds of kilobases in length. The seemingly simple action of DNA loop creation is central in the coordination of many fundamental biochemical processes, the most prominent examples being the regulation of gene expression [68], site-specific recombination [69] and DNA replication [30,70].

**Figure 4:**
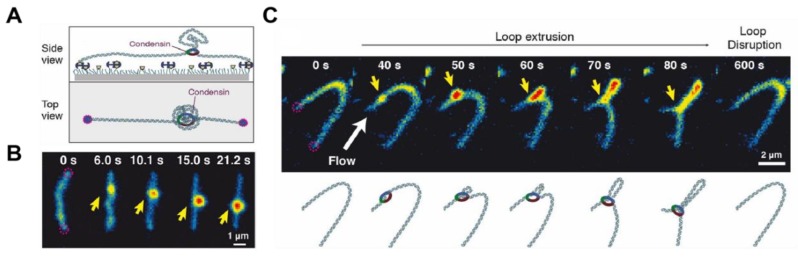
Fluorescence imaging of DNA loop extrusion by condensin. (**A**) Single-molecule assay for the visualisation of condensin-mediated DNA looping. (**B**) Snapshots showing DNA loop extrusion intermediates created by condensin on a SxO-stained doubly tethered λ-DNA. The yellow arrow indicates the location of the loop base. (**C**) Snapshots showing the gradual asymmetric extension of a DNA loop (yellow arrow) on a doubly tethered λ-DNA molecule (reproduced with permission from [73]).

It has been proposed that DNA looping together with DNA supercoiling play critical roles in the spatial organization of chromosomes. Structural maintenance of chromosome (SMC) protein complexes such as condensin and cohesion play key roles in restructuring genomes during the cell cycle [71,72]. How SMC complexes participate in these processes is not completely understood. Single-molecule fluorescence imaging of single DNA molecules have enabled direct, real-time observation of DNA loops extruded from single *Saccharomyces cerevisiae* condensin complexes [73]. The looping process was observed by staining doubly tethered DNA molecules with SxO while being hydrodynamically stretched in a flow of buffer (Figure 4). By monitoring the fluctuations in fluorescence intensity, the authors were able to determine that condensin-induced loop extrusion occurred asymmetrically with an average rate of 0.6 kilobase pairs/second. This finding is in stark contrast to all proposed models of loop extrusion by two linked motor domains. The authors rationalised this unexpected mechanism by proposing that one site in the condensin complex is stably bound to the DNA, while its motor site translocates along the same DNA.

The details of DNA looping and bending at the nanometre level cannot be studied using intercalating or groove-binding dyes that sparsely interact with dsDNA in a sequence-independent manner. To obtain spatial resolution at such length scales, fluorophores can be installed at specific locations of DNA (or protein) to image specific regions or structural domains of a protein or DNA molecule. SmFRET is widely used to study the evolution of nanometre-length scale conformational changes of protein–DNA and protein–protein complexes at the single-molecule level. Not only is FRET a direct imaging technique; it can also measure distances between fluorophores by the extent of non-radiative energy transfer between two fluorescent dye molecules (donor and acceptor). Development of smFRET assays in the Ha and Kim labs have enabled detailed insight into the thermodynamic and kinetic behaviours of DNA bending and loop formation, with the aim to understand the poor ligation efficiencies observed in ensemble cyclisation methods [74,75,76]. In these smFRET experiments, fluorophores (Cy3 and Cy5) placed at known positions on dsDNA molecules with complementary overhangs (sticky ends) are immobilised onto the glass coverslip. Fluorescence signals are observed when molecules are trapped in the looped state due to base pairing between the sticky ends. Looping and unlooping of DNA lead to fluorescence intensity fluctuations, where low FRET signals correspond to the unlooped state and high FRET signals correspond to the looped state. Subsequently, the looping probability density (J factor) can be extracted from the looping rate and annealing rate between the two disconnected overhangs. By probing different intrinsic curvatures, the authors were able to demonstrate that the J factor is sensitive to the intrinsic shape of the DNA [75,76]. Moreover, the role of DNA looping in facilitating protein diffusion and intersegmental transfer can be directly addressed using this strategy. In protein-induced fluorescence enhancement (PIFE) a fluorescent dye on the DNA is placed in proximity to a protein binding site. When the protein binds to this site, it can enhance the fluorescence intensity of the adjacent dye via PIFE. A DNA-binding restriction enzyme was used to demonstrate the feasibility of the assay, defining its target search mechanism on DNA through loop-mediated intersegmental transfer [76].

## 3. Visualisation of Single-Stranded DNA

Single-stranded (ss) DNA is an important intermediate in the fundamental biochemical processes responsible for the maintenance of genome integrity. To date, there is a lack of molecular tools that allow direct visualisation of ssDNA using single-molecule fluorescence microscopy. This gap in the single-molecule toolbox is largely due to the inability to reliably produce long segments of ssDNA and the unavailability of fluorescent probes that directly bind ssDNA with high selectivity. Moreover, the physical properties of naked ssDNA do not allow it to be readily stretched out under easily accessible experimental conditions, unlike dsDNA. In order to stretch ssDNA to a reasonable length, a force higher than at least 5 pN is required, which is not practical with the laminar flows typically used in fluorescence-based single-molecule assays [77].

In an effort to overcome these challenges, the properties of single-strand-binding proteins have been exploited. In this context, binding of single-strand-binding proteins (SSB) to ssDNA enables stretching and visualisation of ssDNA during single-molecule fluorescence imaging. Bell et al. [78] generated ssDNA molecules using DNA from bacteriophage λ that had been biotinylated at the 3′ ends, alkali-denatured, neutralised with buffer and subsequently saturated with fluorescently labelled *Escherichia coli* SSB. Using this strategy, the authors were able to directly monitor the nucleation and growth of RecA filaments on SSB-coated ssDNA one molecule at a time. Gibb et al. [79] furthered this experimental strategy by incubating ssDNA substrates produced from rolling-circle amplification to produce very long ssDNA curtains anchored to chromium barriers. This approach has allowed researchers to investigate questions related to protein–ssDNA interactions, especially those critical in DNA-repair pathways. Using this experimental setup, De Tullio et al. [80] and Kaniecki et al. [81] watched individual Srs2 helicases disrupt DNA-repair intermediates formed by replication protein A, Rad51 and Rad52.

## 4. Studying Biological Processes on DNA: DNA Replication

The classic textbook view taught in biochemistry classes depicts biochemical reactions as being defined and calculated, resulting in discrete complexes that are largely deterministic. Most biochemical pathways require the involvement of multiple protein components, typically forming large complexes which progress through various catalytic states and conformations. Novel fluorescent single-molecule imaging techniques have made significant headway into challenging these oversimplified views of biochemical reactions, particularly during DNA synthesis.

**Figure 5:**
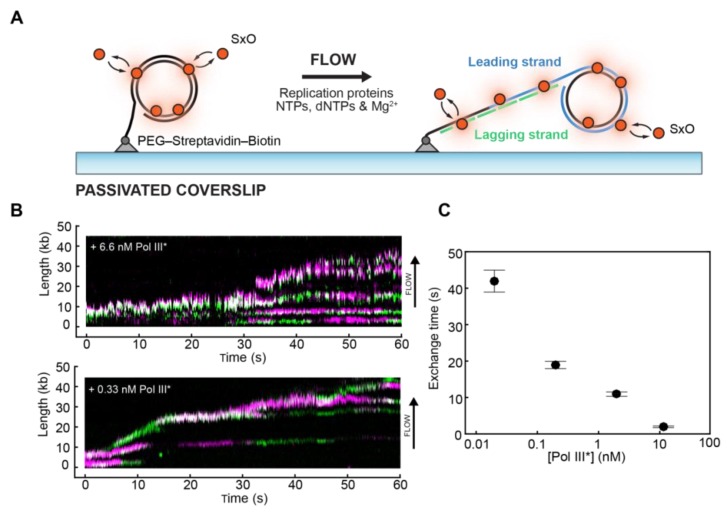
Visualisation of replisome dynamics during DNA replication. (**A**) Cartoon representation of the single-molecule rolling-circle replication assay. A 5′-biotinylated circular DNA molecule is coupled to the surface of a passivated microfluidic flow cell through a streptavidin linkage. Addition of replication proteins and deoxyribonucleotide triphosphates (dNTPs) initiates DNA synthesis. The DNA products are elongated hydrodynamically by flow, labelled with SxO and visualised using fluorescence microscopy. (**B**) Rapid and frequent exchange of Pol III* (holoenzyme lacking the β_2_ sliding clamp) is concentration-dependent. Representative kymographs of the distributions of two different fluorescently labelled Pol III* (magenta and green) on individual DNA molecules at different concentrations. (**C**) Exchange times as a function of Pol III* concentration (reproduced with permission from [85]).

Single-molecule approaches have provided a means to simultaneously observe the proteins and DNA during DNA synthesis, furthering our understanding of the events at the replication fork. Efficient visualisation of DNA replication at the single-molecule level using in vitro reconstituted replisomes has largely been achieved using an assay based on rolling-circle amplification of DNA developed in the van Oijen and O’Donnell laboratories [82,83,84,85,86,87]. The construction of the template yields a forked circular DNA which is biotinylated at the 5′ tailed end (Figure 5A). The template is immobilised onto a functionalised glass coverslip, initiating replication through addition of proteins and flow-stretching the elongating DNA product. During normal conditions, model replisomes readily generate long segments of newly replicated DNA, hundreds of kilobase pairs long. These long DNA products can be easily visualised via real-time TIRF microscopy by staining the dsDNA with SxO or YOYO-1.

Until recently, the replisome was thought to be in a stably associated form during the entirety of the replication process. During processive replication, a single set of DNA polymerases are reused for the recurrent synthesis of many Okazaki fragments. Such efficient recycling of replicative components has been convincingly demonstrated by in vitro bulk-phase biochemical experiments [88,89]. Single-molecule fluorescence imaging of T7 bacteriophage replisomes has shown that DNA polymerases undergo rapid and frequent exchange in and out of replisomes [90,91]. The discrepancy with the bulk-phase literature can be explained through the reduction of the exchange rate of polymerases in and out of the replisome under the highly dilute conditions used in these original experiments. In the cell, the polymerase exchange mechanism ensures the continuing supply of polymerases. Recent work conducted in independent laboratories using fluorescently labelled DNA polymerases have in fact confirmed that DNA polymerases in the gram-negative *E. coli* replisome are frequently exchanged at the replication fork [85,92]. These studies were able to extract the dwell times of individual polymerases at the replication fork, as well as identifying the dependence of exchange frequency on polymerase concentration (Figure 5B,C). Furthermore, single-molecule studies in other living cells have seen the exchange of DNA polymerases in the gram-positive bacterium *Bacillus subtilis* in being recruited and released from active replisomes [93]. Collectively, these observations support a view of a highly dynamic replisome, one that allows both recycling and exchange dynamics of components.

Another widely accepted concept regarding the replisome involves the coordination of leading- and lagging-strand synthesis for faithful replication. The mechanism behind how the enzymatically slow steps of primer synthesis and lagging-strand polymerase loading coordinate with the high rate and continuity of the leading-strand polymerase are largely speculative. Many contradicting models have emerged in the literature to explain such a mechanism of coordination [30,82,88,94,95,96,97,98]. Recent studies by Duderstadt et al. [99] propose that replication can occur via multiple coordination pathways and is regulated by both ssDNA looping and leading-strand synthesis pausing. Another proposed mechanism suggests that leading-strand synthesis is not delayed during priming, but rather that the leading-strand polymerase synthesises slower than the lagging-strand polymerase [70]. Conformation of the lagging-strand template was investigated using DNA FRET pairs observing the formation of priming loops on the lagging strand. This implied that DNA synthesis can continue without interruption and that primers can be synthesised parallel to DNA polymerisation. In *E. coli*, the separation of the helicase and primase functionality into two distinct proteins further complicates the replisome and hence grants access to a broader range of possible coordination mechanisms. The possibility that the replisome does not coordinate leading- and lagging-strand synthesis, but instead consists of independent replication proteins acting in kinetically discontinuous replication has been further investigated by Graham et al. [100]. Experimental evidence provided by visualising DNA molecules stained with SxO establishes that individual trajectories of both leading- and lagging-strand Pol III cores display comparable synthesis rates stochastically scattered with pauses. Additionally, priming frequency is inversely correlated to DnaG concentration, but has no effect on the synthesis rates of either polymerase. The replisome is described as containing individual components that accommodate the discontinuity of lagging-strand synthesis by slowing down. This stochastic model of DNA replication makes it more likely that a replisome can progress past damage on the template DNA, an area of study that remains to be investigated.

Novel single-molecule fluorescence tools have been developed to examine eukaryotic DNA replication in real time. While real-time single-molecule fluorescence assays using entirely reconstituted eukaryotic replisomes have not yet been achieved, investigation using cell-free extracts derived from *Xenopus* eggs have yielded important biochemical insights [101,102,103,104]. By visualising individual DNA molecules replicated by replisomes assembled from undiluted *Xenopus* extracts, Yardimici and co-workers [105] demonstrated that no physical association is required between sister replisomes during elongation. These observations suggested that replisomes emanating from the same origin can function independently during DNA replication. Replication was detected after partial completion of the replication reaction by two independent means: the use of fluorescently labelled anti-digoxigenin to detect incorporation of digoxigenin–dUTP and SxO to stain dsDNA. Individual proteins bound at the replication fork could not be visualised due to the background arising from the high concentration of the fluorescent protein needed to compete with the extract’s endogenous protein. To overcome this limitation, a novel imaging approach termed PhADE (photoactivation, diffusion and excitation) was developed using photo-switchable fluorescent probes to selectively observe those molecules bound to a tethered substrate, allowing imaging of single molecules at previously inaccessible concentration regimes [106]. PhADE exploits the surface confinement of DNA to locally photoactivate DNA-bound molecules. After photoactivation, diffusion of unbound molecules from the detection volume rapidly reduces background fluorescence. To demonstrate this approach, the authors labelled the eukaryotic DNA replication protein flap endonuclease 1 (Fen1) and added it to replication-competent *Xenopus* egg extracts. PhADE imaging of high concentrations of the labelled protein (2–4 μM) revealed dynamics of Fen1 on newly replicated DNA.

Using highly purified proteins, Ticau et al. [107] developed a single-molecule loading assay to understand stoichiometry and dynamics during helicase loading and activation in eukaryotes. Surface-tethered DNA molecules were fluorescently labelled with organic dye molecules. One or two fluorescently labelled licensing factors (i.e., MCM2–7 and Cdc6, or MCM2–7 and Cdt1) were added in the presence of ATP and the protein binding and unbinding was observed in real time by colocalising the fluorescence intensity from DNA with the proteins of interest. By monitoring the arrival and departures of these proteins relative to one another, the short-lived intermediate states could be elucidated. Furthermore, the stoichiometry of the different factors could be derived by photobleaching the labelled proteins. These studies revealed important steps in the pathway, such as the recruitment of MCM2–7 one hexamer at a time [108,109]. Subsequently, by monitoring ORC dynamics, the authors showed that one ORC complex directs the loading of both helicases in each double hexamer [108,109,110]. The findings reveal the complex protein dynamics that coordinate helicase loading and indicate that distinct mechanisms load the oppositely oriented helicases that are central to bidirectional replication initiation.

The ability to monitor individual multi-protein complexes on DNA during biological reactions and observe the short-lived intermediate states has challenged our views of multi-protein systems. In particular, the principles by which they operate are not as linear and deterministic as previously suggested. Rather, these multi-protein systems may utilise many different pathways to achieve the desired outcome. This plurality in behaviour has also been observed more recently in studies with DNA-repair proteins, with the observation of stochasticity and plasticity governing how proteins recognise mismatched nucleotides using ATP to stably link themselves to the DNA in order to facilitate interactions with different proteins [111,112]. The various recent single-molecule studies that characterise the way in which multi-protein complexes reach a desired biological outcome seem to suggest that complex biochemical pathways are largely dictated by the kinetic and thermodynamic boundary conditions encountered along the way. Thus, these multi-protein complexes display a variety of behaviours that cannot be described as individual, well-defined pathways, but instead need to be thought of as complex free-energy landscapes along which reaction coordinates lie.

## 5. Internal Site-Specific Labelling on DNA: Visualisation of Long DNA Molecules

Labelling DNA site-specifically requires incorporation (or attachment) of small molecules or functional groups to DNA that are not native to its structure. These modifications may be achieved chemically or through enzymatic activity. In fluorescence-based approaches, labels are often fluorophores which enable direct detection of DNA, or haptens, which provide secondary binding sites for other functionalised moieties (i.e., biotin- or digoxigenin-conjugated nucleotides). Beyond the scope of single-molecule experiments, labelling DNA in a site-specific manner is of general interest to many scientific disciplines. Thus, many approaches have been devised (reviewed in [113]). In the following section, we describe two unique tools from this diverse molecular toolkit that allow sequence-specific labelling of genomic DNA molecules.

### 5.1. Molecular DNA Combing

Manipulation of DNA is difficult in its natural coiled state, so most single-molecule imaging strategies require confinement of DNA to a physical surface, followed by either mechanically stretching out the DNA, by physically pulling on it or stretching it out in a flow of buffer. Molecular DNA combing has been used as a single-molecule approach to examine chromosomal DNA that has been pulse-labelled with halogenated analogues of thymidine [114], including 5-bromo-2-deoxyuridine (BrdU), 5-iodo-2-deoxyuridine (IdU) and 5-chloro-2-deoxyuridine (CldU). The synthetic halogenated nucleotides are incubated with dividing cells, and then the DNA is isolated. Subsequently, the modified nucleotides within the DNA are fluorescently labelled with antibodies. Individual naked DNA molecules are then visualised by uniformly stretching them onto a silanised microscope coverslip through the action of a receding air-water meniscus. Molecular DNA combing is a powerful approach to accurately monitor both spatial and temporal changes during DNA replication of genomes with single-molecule resolution. Classical techniques used to identify origins of replication include PCR and 2D gel electrophoresis, which are able to detect changes in DNA intermediates, such as bubbles and replicating forks. On the other hand, molecular DNA combing allows precise localisation and quantification of these DNA intermediates. Therefore, it comes as no surprise that molecular DNA combing has been used to monitor DNA replication in a variety of organisms from bacteria all the way up to higher eukaryotes [115,116]. This technique is well equipped to measure origin firing and DNA replication kinetics, as well as genomic rearrangements. Furthermore, molecular DNA combing allows for high-resolution analysis of repetitive sequences, which are often difficult to investigate with DNA sequencing techniques. Importantly, molecular DNA combing can be used to monitor replication defects caused by gene mutations or by chemical agents that induce replication stress or replication roadblocks [117]. In comparison to other methods that stretch DNA, molecular DNA combing is a conceptually simple and reliable method to visualise genomic DNA without the need for tedious genetic manipulation.

Investigation of genomic DNA by molecular DNA combing using single-molecule fluorescence microscopy was first established in the mid-1990s. Bensimon and co-workers were able to quantify the fork speed, symmetry, origin usage and inter-origin distance of bacteriophage λ DNA [114]. Since then, molecular DNA combing has proved invaluable in studying the dynamics of individual replicons in eukaryotes, mitigating the shortcomings of other techniques such as DNA-chip-based approaches. For instance, using the chip method to analyse duplication of *S. cerevisiae* genomes [118], the average rates of replication fork progressions and origin efficiencies were obtained. Using molecular DNA combing, analysis of chromosome VI revealed that replication origins, although well-defined, fired stochastically with no apparent correlation between adjacent origins [119]. While molecular DNA combing has bridged the technological gap between the examination of gross chromosomal abnormalities and sequence-specific alterations [120,121,122], reliable fibre analysis has been typically restricted to molecules of 200–500 kilobase pairs in length. Advancements by Kaykov et al. [123] have been able to substantially improve the procedure to analyse entire chromosomes in fission yeast and 12-megabase fragments from human cells. This technical advancement has led to detection of previously unseen origin clusters in human cells. Furthermore, it revealed that origins in human cells fire stochastically during replication, forming clusters of fired origins. Molecular DNA combing has also been applied to study protein binding of DNA-processing enzymes. Binding of the bacterial chromosomal initiation protein DnaA has been observed on combed genomic DNA from *E. coli* [124]. However, it was not known if DnaA was bound specifically. More recently, Gueroui and co-workers were able to visualise transcription of combed DNA [125]. The transcription activity of T7 RNA polymerase occurred when DNA was stretched close to normal length, but not when overstretched to ~150% the normal contour length. Together, these results open the possibilities to study single enzymes on combed DNA by single-molecule imaging.

### 5.2. Optical Mapping

Optical mapping was developed to create high-throughput, high-resolution genomic maps that contain information about the structure of an organism’s genome. Fundamentally a single-molecule approach, optical mapping requires the mapping of several overlapping DNA molecules to build a physical genomic map. Like mapping roads to depict structural information of a location without needing to detail each individual home, genome mapping can be a powerful tool for understanding variations of large pieces of rearranged or altered genomic DNA. Since their initial development [19], genome-wide optical maps have contributed heavily to establishing structural variations and rearrangements, scaffolding and validating overlapping DNA segments for several large sequencing projects [126,127,128]. Several platforms have transitioned to labelling genomes relying on sequence-specific modification of DNA at short target sites. DNA ‘tagging’ is achieved through enzymatic modification of specific target sites, which are imaged to give a unique overall genomic structure. Typically, three classes of enzymes are utilised to modify DNA at specific sites. These include restriction enzymes, nicking enzymes and DNA methyltransferases (MTases) [129]. Commonly, enzymes such as methyltransferases are used to sequence-specifically transfer a methyl group to the cytosines and adenines in DNA. Most MTases use cofactor S-adenosyl-L-methionine (AdoMet) as a methyl source. This natural methylation reaction can be expanded to a variety of reactions using synthetic cofactor analogues. Used in conjunction with modified AdoMet substrates, these MTases can be used to covalently bind other chemical moieties to specific genomic sequences.

Two labelling strategies that exploit this specific activity are the sequence-specific methyltransferase-induced labelling of DNA (SMILing) and methyltransferase-directed transfer of activated groups (mTAG). Neely et al. [130] used the mTAG approach to densely label DNA with fluorophores and construct optical DNA maps via fluorescence microscopy from individual DNA molecules deposited on a surface. However, this approach resulted in low labelling efficiency, as a consequence of slow amino-to-NHS ester coupling kinetics. In efforts to increase fluorophore coupling efficiencies and reduce the fold coverage required to build reliable maps, copper-catalysed azide-alkyne cycloaddition was explored [131]. By changing the coupling chemistry Vranken and co-workers achieved a labelling efficiency of ~70%, from 50% and a labelling density of one label every 500 base pairs. This improvement allowed for a true single-molecule map of the DNA sequence to be produced, bridging the gap between typical sequencing experiments and long-range information obtained from traditional optical mapping. In another approach, a one-step chemo-enzymatic reaction was used to covalently bind fluorophores to DNA at the four-base recognition site of Mtase M.TaqI to accurately genotype genomes of λ and T7 bacteriophages from a background phage library [132].

Ultimately, the use of methyltransferase-based labelling depends on the availability or ease of synthesis of modified AdoMet analogues, which are not widely available. However, DNA labelling using methyltransferase enzymes is rapid, highly efficient and can potentially be combined with any fluorescence imaging platform to generate high-resolution DNA maps. Labelling can be directed to four- or six-base target sequences, allowing the mapping of DNA at a labelling density that suits the application. This method provides a facile route for screening and typing of various organisms and has potential applications in epigenetics of various organisms.

## 6. Commercial Applications of Single-Molecule Sequencing

Many modern biotechnologies are based on our growing understanding of DNA structure and function. A range of DNA-based laboratory techniques are used to study whole genomes for medical genomics research and diagnostics. Sequencing technologies are used in a wide range of applications of several different kinds of genomic testing, from prenatal diagnostics to diagnosing rare diseases, hereditary forms of cancer and pharmacogenetics [133]. Commercially, only a few single-molecule sequencing technologies are available which have successfully been used in several scientific disciplines. Nanopore sequencing, commercialised by Oxford Nanopore Technologies, utilises electrophoresis to drive single-stranded RNA or DNA across lipid layers in large ion channels. Another single-molecule sequencing technology commercialised by Pacific Biosystems is single-molecule real-time (SMRT) DNA sequencing. SMRT DNA sequencing uses fluorescent phospholinked nucleotides, in which each of the four nucleotides are labelled with a different coloured fluorophore. The resulting fluorescent signal is monitored in nanoscale cylindrical cavities ~100 nm in diameter and height, called zero-mode waveguides (ZMWs). A ZMW is an optical waveguide that directs light energy into a well that is smaller than the wavelength of the illuminating light. This allows detection of single fluorophore molecules close to the bottom of the ZMW at high concentrations. As DNA synthesis occurs at the rate of the polymerase, nucleotide incorporation is counted and classified based on the fluorescent signals produced after each nucleotide incorporation. Incredibly long read-outs exceeding 10,000 base pairs in length can be generated, which is useful for de novo genome assemblies. Apart from DNA sequencing, ZMWs have been exploited for single-molecule RNA sequencing and epigenetics [134].

While SMRT DNA sequencing allows for long sequence read-outs, a critically limiting step is the efficient loading of small quantities of long DNA molecules into ZMW confinements. This is due to the substantial sampling time needed for the polymerase-bound DNA to bind the ZMW and entropic barrier to entry under diffusive conditions, which favour the escape of longer DNA from the ZMW over short DNA [135]. In an effort to overcome these limitations, Larkin et al. [136] used voltage-induced DNA loading into ZMWs equipped with nanopores. The authors found that this combination improved binding efficiency by five orders of magnitude and that DNA loading was nearly length-independent. While we have made remarkable progress in understanding the capabilities of single-molecule sequencing technologies over the past decade, several key challenges still need to be solved before these technologies become cost-effective and widely available in medical clinics and research laboratories.

## 7. Outlook

It is clear that the single-molecule technologies developed to monitor the dynamic properties of individual DNA molecules in isolation and during biochemical reactions have outgrown the constraints of classical techniques used in structural biology. The development of a diverse molecular toolkit to study DNA with single-molecule resolution required the advancement of new technologies for detection and analysis. While significant, these efforts have rewarded us with valuable insights, intangible when only measuring an averaged population or studying static images. As experimentalists of different backgrounds increasingly engage with single-molecule biophysics, new methods to visualise DNA in increasingly larger, more complex systems will be developed and older ones refined. The ability to visualise individual DNA molecules during DNA replication has already challenged textbook views on the stability of replisomes, demonstrating that these multi-protein complexes are not static entities, and rather that they behave in a highly dynamic fashion. The use of fluorescent-based probes described in this review have advanced to a point in which the questions that can be answered appear inexhaustible. However, the application of these tools to visualise ssDNA in real time is comparatively limited. Development of new chemical and protein-based probes to visualise ssDNA will offer the potential for new insights into the mechanisms associated with fundamental processes such as DNA replication, repair and recombination. Moreover, further development of fluorescence-based probes to watch DNA and its intermediates within living cells will be required to bridge the gap between in vitro experiments and those carried out on living cells.
